# Transarterial chemoembolization for early stage hepatocellular carcinoma decrease local tumor control and overall survival compared to radiofrequency ablation

**DOI:** 10.18632/oncotarget.12921

**Published:** 2016-10-26

**Authors:** Arnaud Hocquelet, Olivier Seror, Jean-Frédéric Blanc, Nora Frulio, Cécile Salut, Jean-Charles Nault, Hervé Trillaud

**Affiliations:** ^1^ Department of Diagnostic and Interventional Imaging, Hôpital Saint-André, Centre Hospitalier Universitaire de Bordeaux, Bordeaux, France; ^2^ EA IMOTION (Imagerie Moléculaire et Thérapies Innovantes en Oncologie) Université de Bordeaux, Bordeaux, France; ^3^ Department of Radiology, Hôpital Jean Verdier (Assistance Publique-Hôpitaux de Paris), Bondy, France; ^4^ Department of HepatoGastroenterology and Digestive oncology, Hôpital Saint-André, Centre Hospitalier Universitaire de Bordeaux, Bordeaux, France; ^5^ Inserm, Génomique Fonctionelle des Tumeurs Solides, Paris, France

**Keywords:** chemoembolization, therapeutic, radiofrequency ablation, carcinoma, hepatocellular

## Abstract

**Background & Aims:**

To compare treatment failure and survival associated with ultrasound-guided radiofrequency ablation (RFA) and trans-arterial chemoembolization (TACE) for early-stage HCC in Child-Pugh A cirrhosis patients.

**Methods:**

122 cirrhotic patients (RFA: 61; TACE: 61) were well matched according to cirrhosis severity; tumor size and serum alpha-fetoprotein. TACE was performed in case of inconspicuous nodule on US or nodule with “at risk location”. Treatment failure was defined as local tumor progression (LTP) and primary treatment failure (failing to obtain complete response after two treatment session). Treatment failure and overall survival (OS) were compared after coarsened exact matching. Cox proportional model to assess independent predictive factors was performed.

**Results:**

No significant difference was seen for baseline characteristics between the two groups. Mean tumor size was 3cm in both group with 41% HCC>3cm. Treatment failure rates after TACE was 42.6% (14 primary treatment failures and 12 LTP) and 9.8% after RFA (no primary treatment failure and 6 LTP) *P* < 0.001. TACE was the only predictive factor of treatment failure (Hazard ratio: 5.573). The 4-years OS after RFA and TACE were 54.1% and 31.5% (*P* = 0.042), respectively.

**Conclusion:**

For Child-Pugh A patients with early-stage HCC, alternative treatment as supra-selective TACE to RFA regarded as too challenging using common US guidance decrease significantly the local tumor control and overall survival. Efforts to improve feasibility of RFA especially for inconspicuous target have to be made.

## INTRODUCTION

Hepatocellular carcinoma (HCC) is the fifth most common cancer and the second cause of cancer-related deaths [1]. As recommended by EASL clinical practice guidelines [1] single hepatocellular carcinoma and up to three hepatocellular carcinoma < 3cm should be treated by transplantation, surgery resection (SR) or radiofrequency ablation (RFA). Due to the lack of transplants from cadaveric donors RFA is the first-line treatment for unresectable Child Pugh A HCC patients. Although TACE is recommended only for BCLC B HCC [1], this treatment is the most performed worldwide and it is frequently used for early-stage HCC. Indeed, thirty per cent of RFA can't be performed under ultrasound guidance because of tumor invisibility [2] and 15% of RFA are not performed due to the “high-risk” location of the tumor [3]. Recent progress in imaging guidance as imaging fusion [4, 5] or cone-beam CT [6, 7] and using artificial pleural effusion or ascitis [3, 8], [9] have drastically reduced the RFA infeasibility rate. However these devices are mainly available on specialized tertiary centers, When not available, supra-selective transarterial chemoembolization (segmental or subsegmental tumor feeding embolization) (TACE) [10] is frequently used as first-line treatment [11]. Furthermore several studies concluded that supra-selective TACE allows achievement of long-term survival rates comparable to RFA for early-HCC [12–14]. These studies explained the similar outcome by a less satisfactory effect of RFA on medium tumors (3-5 cm in diameter) and the ability of TACE treating satellite nodules. Indeed monopolar radiofrequency device offered a weak local disease control and complete necrosis for HCC larger than 3cm [15, 16]. RFA technologies have been improved. No touch multi-bipolar RFA offered a larger complete necrosis rate than monopolar devices for medium HCC [16–19] and avoids the need of intra tumorous puncture. So it appeared as more suitable ablative technique for inconspicuous target with ultrasound or medium size HCC on condition to use proper advanced guidance imaging. Thus because main causes of infeasibility of RFA are nowadays resolved, it is of major interest to value if supra-selective TACE can compete with RFA as first line treatment in curative intent for HCC ≤5cm. The aim of this study was to compare the treatment failure (defined as local tumor progression and failing to obtain complete response) and the following overall survival in Child-Pugh A cirrhotic patients after RFA *versus* supra-selective TACE as first line treatment for HCC ≤5cm criteria using coarsened exact matching.

## RESULTS

**Figure 1:**
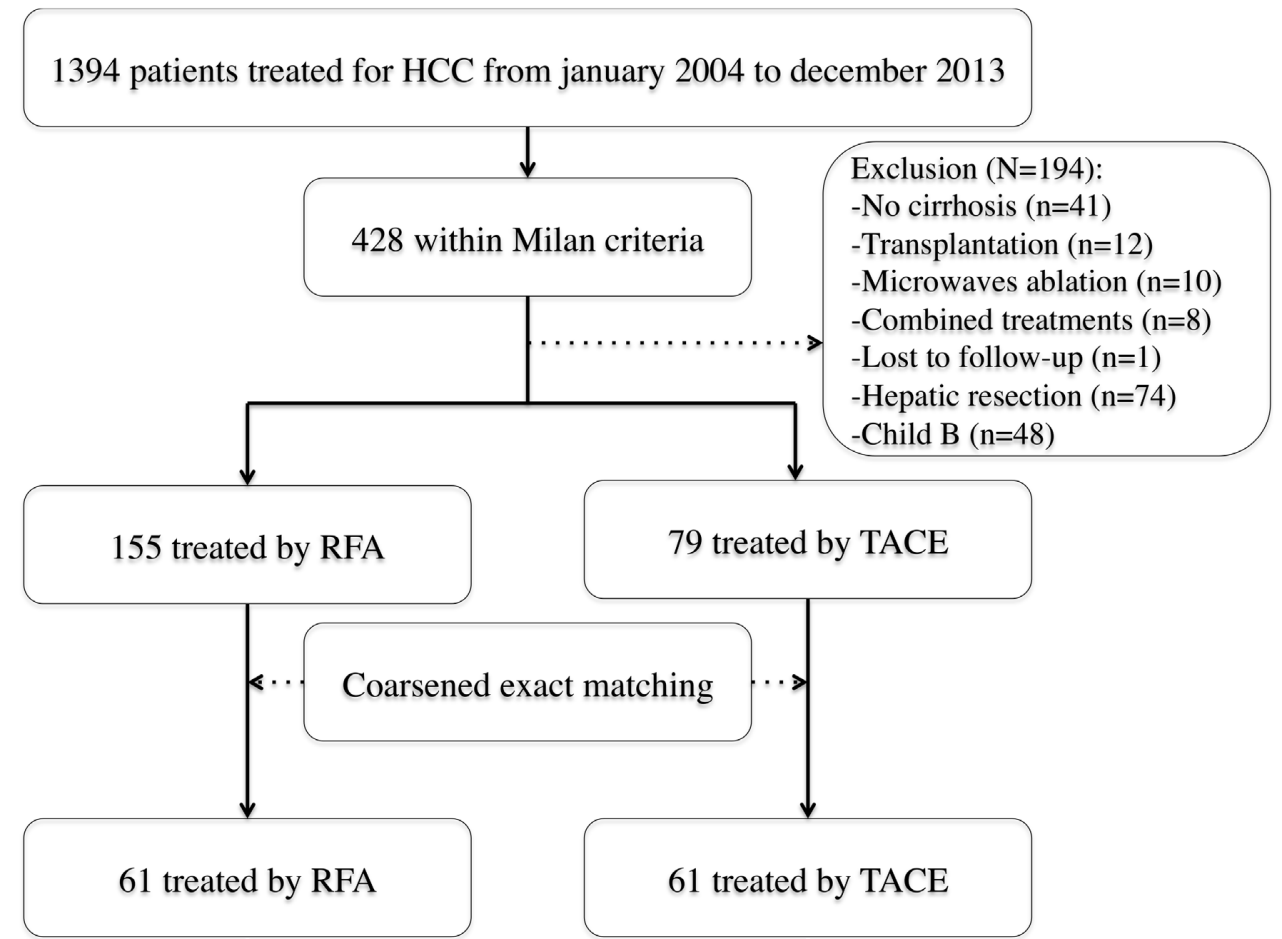
Flow-chart

Among the 234 patients with HCC≤5cm (Figure 1), 122 Child A cirrhotic patients treated either by RFA (*n* = 61) or TACE (*n* = 61) were matched.

### Baseline characteristics of RFA and TACE group

The mean tumor size was 30.2 (±10) mm in RFA group and 31 (±10) mm in TACE group (*p* = 0.399). 41% of patients in each treatment groups had HCC > 30mm. No significant difference between both groups was seen for baseline characteristics and patients were perfectly matched according to tumor size and serum AFP strata (Table 1). TACE was chosen as first-line treatment due to: inconspicuous nodule on US for 33 patients (54%); subcapsular location for 10 patients (16.4%) and “at risk location” for 18 patients (29.5%) [13 near hepatic hilum; 2 near colon; <1 near stomach; 1 near the inferior vena cava and one near a sus-hepatic venous].

**Table 1:** Demographics and clinical characteristics of Child-Pugh A patients who received radiofrequency ablation (RFA) and transaterial chemoembolization (TACE) for hepatocellular carcinoma (*N* = 122)

	RFA group (*n* = 61)	TACE group (*n*= 61)	*P* value
Age**	67 (11)	67 (11)	0.919
**Male** ***n*** **(%)**	50 (82)	47 (77)	0.501
BMI**	27.8 (4.7)	27.6 (5)	0.892
Platelet count (G/L)*	122 (86-162)	121 (81-160)	0.706
**Etiologies**: ∙ **HCV** ∙ **Non-viral** ∙ **Mixed**	13 (21) 42 (69) 6 (10)	14 (23) 40 (66) 7 (11)	0.922
AFP (ng/ml)*	10 (5-42)	10 (4-43)	0.350
**AFP (categorical)** ***n*** **(%)** ***- <10 ng/ml*** ***- 10-100 ng/ml*** ***- >100ng/ml***	30 (49) 19 (31) 12 (20)	30 (49) 19 (31) 12 (20)	1
Tumor size (cm)**	30.2 (10)	31 (10)	0.399
**Tumor size (categorical)** ***n*** **(%)** **-** ***<2 cm*** ***- 2-3 cm*** ***- 3.1-5 cm***	8 (13) 28 (46) 25 (41)	8 (13) 28 (46) 25 (41)	1
**Multiple nodules**: ***n*** **(%)**	14 (23)	19 (31)	0.308

*median (1 and 3 quartiles) and compared with Wilcoxon rank-sum test

** Mean (Standard Deviation) and compared with two sided T-test

Categorical variables are: n (%).

Abbreviations: BMI= Body Mass Index; HCV= Hepatitis C virus; AFP= Alpha-Foeto-protein;

In RFA group, Monopolar device was used for 19 patients (31%) and multipolar devices for 42 patients (69%) (including the 25 patients with HCC > 3cm).

The mean follow-up was 2.7 years (±1.9); the median follow-up was 2.25 years.

Survival status at the end of the study was available for 121/122 patients. One patient was lost to follow-up in RFA group after 5.58 years. The rate of liver transplantation did not differ between the two groups, 13% (8/61) in RFA group and 11% (7/61) in TACE group, *p* = 0.783. The mean and median time to transplantation were 2.01 years (range: 0.42-5.44) and 2.03 years (1-3 quartiles: 0.98-2.8), respectively.

Median hospitalization duration was 2 days (range: 2-6) in RFA group and 2 days (range 2-7) in TACE group, *p* = 0.902.

### Treatments failure and predictive factor

A complete response (CR) was achieved in 100% of patients in RFA group, with 4 patients (6.5%) requiring two ablative sessions. A CR was observed for 47 patients (77%) in TACE group, after one session for 28 patients (59.5%), two sessions for 19 patients (40.5%). The rates of CR and the number of treatment session to achieve it were significantly different between both groups, *p* < 0.001 for both. Eight patients (13.1%) treated by TACE experienced partial response, two (3.2%) stable diseases and four (6.5%) progressive diseases as best treatment response.

Local tumor progression was observed in 9.8% of patients after RFA (6/61) *versus* 25 % of patients after TACE (12/47), *p* = 0.03.

**Figure 2:**
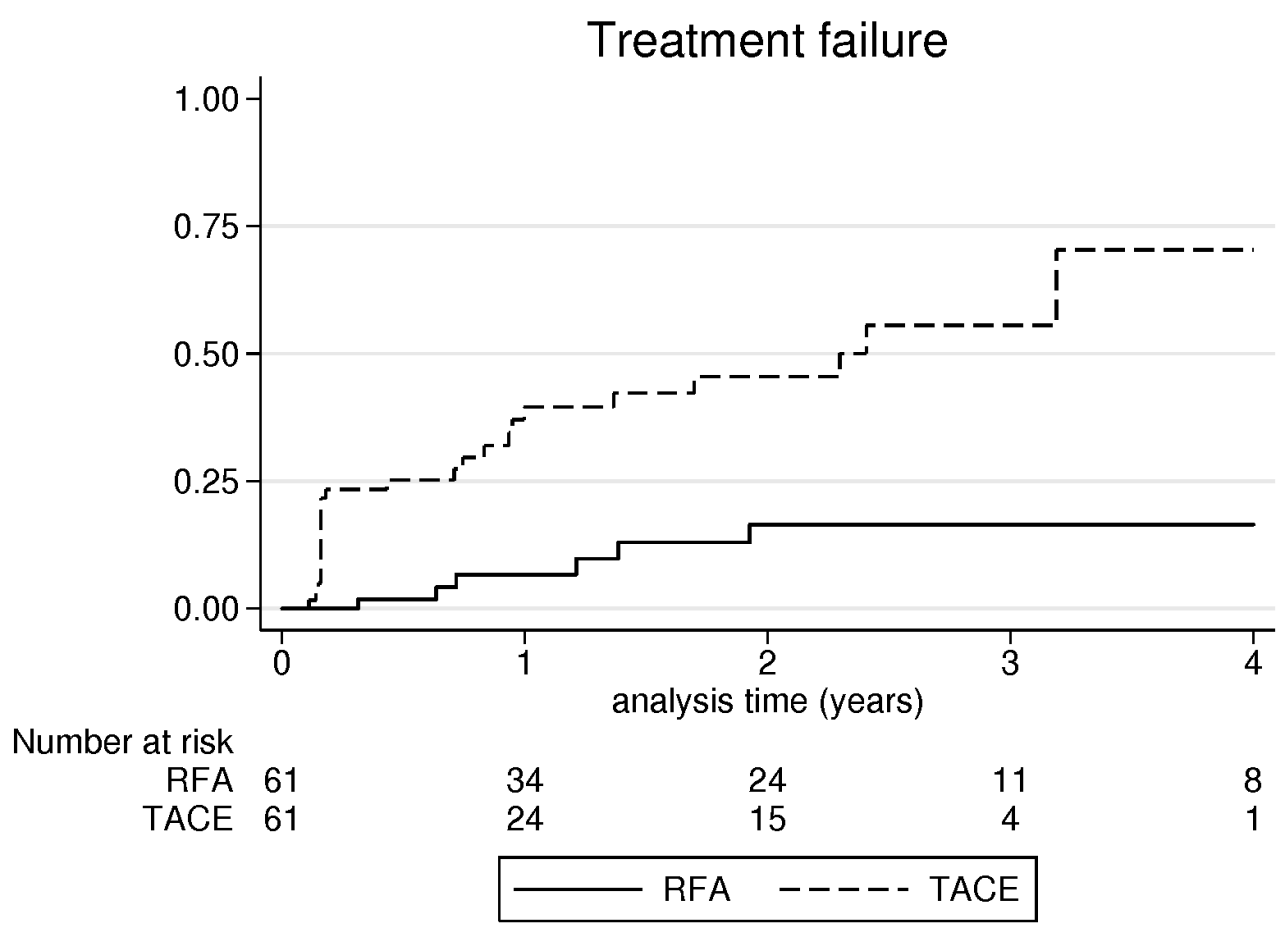
Cumulative incidence of treatment failure after radiofrequency ablation and transarterial chemoembolization

Consequently, the rate of treatment failure (primary treatment failure and LTP) was significantly higher after TACE (42.6%) than after RFA (9.8%), *p* < 0.001 (Figure 2).

According to tumor size, < 2cm; 2-3cm and 3.1-5cm, treatment failure rates for RFA and TACE were respectively, 12.5% (1/8), 10.7% (3/28) and 8% (2/25) *versus* 50% (4/8), 32% (9/28) and 52% (13/25).

In uni and multivariate analysis (Table 2), supra selective TACE was the only predictive factor of treatment failure, with hazard ratio (95% CI): 5.573 (2.281 -13.62), *P* < 0.001.

**Table 2:** Predictive factor of treatment failure (primary treatment failure and local tumor progression)

Variable	Univariate analysis		Multivariate analysis	
	*P*	Hazard ratio (95%CI)	*P*	Hazard ratio (95%CI)
**Age (years)**	0.503	0.988 (0.955-1.022)		
**Sex (Male) (%)**	0.151	0.578 (0.273-1.218)		
**BMI**	0.391	0.968 (0.899-1.042)		
**Platelet count (<100G/L)**	0.517	1.268 (0.618-2.602)		
**Non-Viral hepatitis**	0.227	1.357 (0.827-2.228)		
**AFP>100 ng/ml**	0.250	1.605 (0.716-3.598)		
**HCC>3cm**	0.306	1.453 (0.710-2.974)		
**Multiple nodules**	0.542	1.272 (0.586-2.760)		
**TACE (*vs*** **RFA)**	**<0.001**	**5.573 (2.281 -13.62)**	**<0.001**	**5.573 (2.281 -13.62)**

Abbreviations: BMI= Body Mass Index; ALT= Alanine Amino Transferase; AFP= Alpha-Foeto-protein; HCC= HepatoCellular Carcinoma; RFA= Radiofrequency ablation; TACE= transarterial chemoembolization.

Pathological examination of initially treated tumors performed on explanted livers showed a mean necrosis percentage of 96% (range: 80-100) after RFA *versus* 61.4% (range: 20-100) after TACE (*p* = 0.008). In RFA group with explanted liver examination (*n* = 8), six patients showed complete tumor necrosis, 1 tumor necrosis equal to 90% and one 80%. In TACE group with explanted liver pathological examination (*n* = 7), only one patient showed complete tumor necrosis, one equal to 80%, two 70%, one 50%, one 40% and one 20%.

### Overall survival

**Figure 3:**
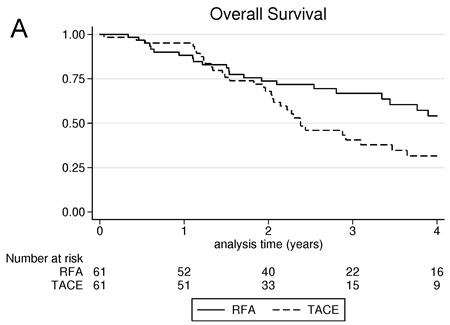
**A**. overall survival comparison between radiofrequency ablation and trans-arterial chemoembolization; **B**. Progression-free survival comparison between radiofrequency ablation and trans-arterial chemoembolization

4-years overall survival for RFA and TACE groups were respectively 54.1% - *versus* 31.5%, with a mean and median overall survival of 4.6 years (95% CI: 3.6-5.5) and 4.9 years (95% CI: 3.6-6.3) in RFA group *versus* 3.7 years (95% CI: 12.8-4.5) and 2.4 years (1.7-3.1) in TACE group, *p* = 0.042 (Figure 3A).

The factors used to predict overall survival are summarized in Table 3. In univariate analysis, primary treatment failure (*p* < 0.001), TACE (*p* = 0.045), multiple nodules (*p* = 0.019) and serum AFP > 100ng/ml (*p* = 0.049) were significantly associated with overall survival. In multivariate analysis with the Cox proportional hazards model, primary treatment failure (Hazard Ratio [95% CI]: 4.163 [2.088-8.298]; *p* < 0.001) and multiple nodules (2.257 [1.261-4.039]; *p* = 0.006) were found to be independent predictive factor associated with overall survival.

**Table 3:** Univariate and multivariate cox analysis for factors associated with overall survival for hepatocellular carcinoma patients treated by radiofrequency ablation (RFA) or transarterial chemoembolization (TACE) (*n* = 122)

Variable	Univariate analysis		Multivariate analysis	
	*P*	Hazard ratio (95%CI)	*P*	Hazard ratio (95%CI)
**Age (years)**	0.922	0.998 (0.973-1.024)		
**Sex (Male) (%)**	0.727	0.891 (0.468-1.696)		
**BMI**	**0.097**	**0.952 (0.899-1.008)**		
**Platelet count (<100G/L)**	0.119	1.537 (0.895 -2.640)		
**Non-Viral hepatitis**	0.527	1.122 (0.656-2.274)		
**AFP>100 ng/ml**	**0.049**	**1.799 (1.00 -3.230)**		
**HCC>3cm**	0.641	1.138 (0.660-1.963)		
**Multiple nodules**	**0.019**	**1.987 (1.122-3.519)**	**0.006**	**2.257 (1.261-4.039)**
**Primary treatment failure**	**<0.001**	**3.641 (1.853-7.154)**	**<0.001**	**4.163 (2.088-8.298)**
**TACE (*vs*** **RFA)**	**0.045**	**1.746 (1.012-3.012)**		

Abbreviations: BMI= Body Mass Index; ALT= Alanine Amino Transferase; AFP= Alpha-Foeto-protein; HCC= HepatoCellular Carcinoma; RFA= Radiofrequency ablation; TACE= transarterial chemoembolization.

### Treatments complications

Two major adverse events occurred after TACE: One treatment-related death in a 79 year-old cirrhotic patient with Alzheimer disease. He died twenty-eight days after TACE due to lung infection without liver failure. The other major complication was a liver failure requiring a prolonged hospitalization > 48 hours. In RFA group the major adverse event was abscess developed on ablation site, in the Couinaud segment 2. The rates of adverse event did not differ between the two groups (*p* = 1, by Fisher exact test).

### Progression-free survival (local or intra-hepatic distant recurrence)

The 4-years progression-free survival for RFA and TACE groups were respectively 29.7% *versus* 3% (Figure 3B) with a mean and median progression-free survival of 2.5 years (95% CI: 1.8, 3.1) and 1.4 years (95% CI: 0.601, 2.7) in RFA group *versus* 1.4 years (95% CI: 1.1-1.7) and 0.95 years (0.6-1.3) in TACE group, *p* = 0.009.

Factors associated with progression-free survival are summarized in Table 4. In univariate analysis, primary treatment failure (*p* < 0.001) and TACE (*p* = 0.011) were significantly associated with progression-free survival. In multivariate analysis with the Cox proportional hazards model, primary treatment failure was the only predictive factor associated with progression-free survival, Hazard Ratio: 3.976 (95% CI: 2.175-7.267), *p* < 0.001. Nineteen patients (31%) in RFA group and 27 patients (44%) in TACE group experienced recurrence beyond Milan criteria: 5 secondary to tumor size (3 after RFA and 2 after TACE); 22 secondary to intra-liver multifocal recurrences (7 in RFA and 15 in TACE); 8 secondary to infiltrative HCC (6 in RFA group and 2 in TACE group); 7 secondary to intra-vascular tumoral extension (1 after RFA and 6 after TACE); 3 secondary to lung (*n* = 1), adrenal gland (*n* = 1) or lymph node (*n* = 1) metastasis (one after RFA and 2 after TACE) and one due to intra-ductal recurrence (1 after RFA and none after TACE).

**Table 4:** Univariate and multivariate cox analysis for predictive factor of HCC recurrence

Variable	Univariate analysis		Multivariate analysis	
	*P*	Hazard ratio (95%CI)	*P*	Hazard ratio (95%CI)
**Age (years)**	0.110	0.983 (0.63-1.003)		
**Sex (Male) (%)**	0.944	0.981 (0.583-1.650)		
**BMI**	0.720	0.992 (0.950-1.036)		
**Platelet count (<100G/L)**	0.232	1.304 (0.843-2.016)		
**Non-Viral hepatitis**	0.227	1.357 (0.827-2.228)		
**AFP>100 ng/ml**	**0.100**	**1.521 (0.911-2.539)**		
**HCC>3cm**	0.707	1.008 (0.709-1.657)		
**Multiple nodules**	0.171	1.385 (0.869-2.209)		
**Primary treatment failure**	**<0.001**	**3.976 (2.175-7.267)**	**<0.001**	**3.976 (2.175-7.267)**
**TACE (*vs*** **RFA)**	**0.011**	**1.753 (1.138-2.701)**		

Abbreviations: BMI= Body Mass Index; ALT= Alanine Amino Transferase; AFP= Alpha-Foeto-protein; HCC= HepatoCellular Carcinoma; RFA= Radiofrequency ablation; TACE= transarterial chemoembolization

## DISCUSSION

In this study, we investigated the local tumor control and survival benefits of RFA and TACE with a coarsened exact matching method. It provided a perfect matching for tumor size and serum AFP strata. When RFA cannot be performed under US guidance due to inconspicuous nodule, TACE appears like an attractive treatment alternative especially for HCC≤5cm, if cone-beam CT, CT-Scan or imaging fusion guidance are not available. However the rate of primary treatment failure (failing to achieve complete response) following TACE reached 23% in our cohort and 25% in Kim et al study [14] that is significantly higher than RFA (no primary treatment failure), *p* < 0.001. Beyond primary treatment failure, RFA can produce supra centimeter safety margin that limit the local recurrence rate [20, 21]. At odds supra-selective cTACE does not seems able to produce safety margin [22] due to the lack of portal vein embolization [23] that lead to a high local recurrence rate (25% in our study) despite primary treatment success. So treatment failure rates reached 42.6% after TACE *versus* 9.8% after RFA (*p* < 0.001). LTP and primary treatment failure are both predictive factor of poor outcome [24] as illustrated by the multivariate cox model selecting primary treatment failure as the main prognostic factor of OS (HR: 4.163) and PFS (HR: 3.976). Despite a high mean tumor size (3cm in each group) with 41% of patients with tumor > 3cm, we did not observed an increase of treatment failure using RFA for HCC > 3cm (around 10%) while Kim et al [15] and Cartier et al [19] reported a LTP rate > 70% for medium size HCC using Monopolar RFA. This excellent local tumor control for 3.1-5 cm HCC is explained by using multipolar devices that offer a larger and more homogeneous necrotic area [16, 17, 25–27] than monopolar devices even using overlapping technic [28]. Several authors [15, 29] tested the combination of monopolar RFA with TACE for HCC < or = 5 cm. Compared with RFA alone, improvement of LTP have been reported only for tumor > or = 3 cm in diameter. This strategy could be also interesting for tumor inconspicuous at unenhanced imaging (US or CT).

The 4-years overall survival after RFA and TACE were similar to previous publication studying western cirrhotic patients [30, 31] but at odds to previous eastern studies [12, 14], RFA offered a better OS than supra-selective TACE for HCC≤5cm (P = 0.042). The better OS and PFS provided by RFA compared to TACE could be explained par the lower rate of treatment failure but also by the higher rate of complete pathological tumor necrosis provided by RFA. Indeed on the explanted liver pathological examination we found that 6/8 (75%) tumor treated by RFA were completely necrosis while only 1/7 (14.2%) tumor treated by TACE showed complete necrosis. Allard et al [32] emphasized a clear benefice of a pathological tumor necrosis higher than 90% after TACE on survival after liver transplantation or liver resection. On the same line, Seror et al [17] published that pathological complete necrosis is achieved in more than 90% of cases using no touch multibipolar RFA while the rate of pathological complete necrosis is around 60% after TACE [33]. Moreover, recently drug-eluted beads TACE (DEB-TACE) appears as the main alternative to Lipiodol-TACE with a better pharmacological profile but without translation in better tumor response or survival in multicentric propective trials [34, 35].

Considering the better local tumor control leading to better OS and PFS offered by RFA compared to TACE, RFA should be the standard treatment used as first-line. In case of inconspicuous nodule, TACE should be considered, only when the tumor still unnameable to RFA using advanced technologies for ablation like multibipolar RFA and or for imaging guidance like CT, US fused with CT or MRI or Cone-Beam CT or guidance Indeed Cone-Beam CT since using these techniques 100% primary treatment success has been reported in preliminary reports [6, 7].

Nevertheless, our study has several limitations. The main limitation is the retrospective design although we used coarsened exact matching to avoid selection bias and none patient was lost to follow-up at 5 years. In our study, HCC treated by TACE were mostly inconspicuous on US and or in challenging location for electrode placement. Although this characteristic in our knowledge has never been associated with more aggressive natural tumor grow pattern, it is possible that in that circumstance the trickier radiofrequency needle placement even assisted with advanced imaging-guidance, leads to a higher rate of local tumor progression compared to easier radiofrequency treatment.

No systematic pre-treatment tumor biopsies are performed so tumor differentiations are not known, and cannot be included in matching model.

Despite a high complete response rate (77%), supra-selective TACE is associated with a higher treatment failure (local tumor progression and primary treatment failure) and lower overall survival than RFA using mainly multipolar device. In case of inconspicuous nodule≤5cm, all efforts have to be made using proper technologies (ablation and guidance) to perform RFA rather than TACE.

## PATIENTS AND METHODS

The study protocol conforms to the ethical guidelines of the 1975 Declaration of Helsinki and was approved by the institution's human research committee. Informed consent was not necessary for this retrospective analysis of our data.

### Patients

Patients’ data were collected from a prospectively maintained and computerized database recording: age, sex, cirrhosis etiology, Child score, Platelet count, serum α-fetoprotein level (AFP), number and size of HCC, treatment, results of pathological radiological and pathological examinations. We included all consecutive cirrhotic, Ecog-0 and child-Pugh A patients with single HCC ≤ 5cm or ≤ three nodules ≤3 cm unsuitable for surgery, without extrahepatic metastasis (early-stage HCC), treated by first line RFA or supraselective TACE from January 2004 to December 2013 according the decision of local tumor boards. Additional criteria were: (i) Child-Pugh A; (ii) no other cancer. Exclusion criteria were: (i) Child-Pugh B; (ii) lost-to-follow-up before the first imaging control; (iii) combined treatment (RFA plus TACE or RFA plus surgery); (iv) treated by microwaves ablation; (v) ill-defined tumor. Then patients were matched in two groups according to treatment type: (i) RFA; (ii) TACE according to demographic data, surrogate marker of cirrhosis severity, serum AFP and tumor characteristics.

### Diagnosis of HCC

All patients were cirrhotic. Cirrhosis was histologically proven for 55 patients (45%), and based on liver stiffness, imaging and blood sample analysis for the 67 remaining patients (55%). Non-invasive criteria of the European Association for the Study of the Liver (EASL) were used to diagnose HCC in cirrhotic patients [1]. Diagnosis was performed on multiphase liver MRI or CT-Scan. Nodule was diagnosed as HCC if it was hypervascular in the arterial phase with washout in the portal venous or delayed phases (*n* = 83, 68%). Tumor biopsies with pathologic confirmation were performed for patients who did not meet the non-invasive diagnostic criteria (*n* = 39, 32%).

### Radiofrequency ablation

All RFA procedures were performed percutaneously under general anaesthesia. Real-time ultrasound (US) with a 4-MHz probe was chosen as guidance modality for all patients. Five senior interventional radiologists (at least five years of experience) performed RFA using one of the following devices: monopolar expandable Boston LeVeen ^™^ needles (RF 3000 Boston Scientific Corporate^®^), or multipolar internally cooled-tip CelonProSurge™ (CelonPOWER System OLYMPUS Medical^®^) (available in our center since 2006) [18]. The device was chosen based on the operator's expertise, the tumour shape, size, location and vascular proximity. Operators used multipolar device for ≥ 3cm and in case of vascular proximity. The thermal ablation was performed according to the manufacturer's instructions. Real-time procedure control of RFA was performed with ultrasound examination.

### TACE procedure

The same interventional radiologist performing RFA have performed supraselective TACE (at least five years experience). Portal vein permeability was checked by ultrasound examination before TACE. The transfemoral approach was carried out under local anaesthesia using 4-Fr angiographic catheters. The coeliac and hepatic arteries were catheterized with Cobra or Simmons 4-Fr (Terumo); next segmental and subsegmental tumor feeding arteries was catheterized using micro-catheter PROGREAT (Terumo) 2.8-Fr. An emulsion of 10ml iodized oil (Lipiodol; Andre Guerbet, France) and doxorubicin hydrochloride (50mg in 10 ml) was infused through the feeder vessels. Then embolization was performed using a mixture of gelatin sponge particles and contrast material until reaching a stasis flux.

### Treatment choice

Treatments were decided upon in a multidisciplinary team meeting and the treatment option was chosen based on guidelines [1]. For early-stage HCC unsuitable for surgery, RFA was the first-line treatment. Before that protective manoeuvre as hydrodiscection and/or advanced guidance technologies as fusion US-CT or MR became routinely used or available in our center we preferably chose TACE for inconspicuous nodule on US or nodule with “at risk location” (near gallbladder, bile duct or gastro-intestinal tract). In case of local tumor progression, if HCC was seen on US examination RFA was the first treatment choice, if not TACE was performed.

### Patient follow up

Oncologic follow-up was performed with MRI (or CT-scan in case of contra-indication of MRI) at one month and then each three months for the liver and by chest CT-scan every six months. For TACE, the one-month follow-up consisted in an association of liver MRI and Thoracoabdominal unhanced-CT-scan to evaluate the tumor iodized oil labeling.

### Study endpoint

#### Treatment failure

The main endpoint of the study was to compare treatment failure rates defined as primary treatment failure or local tumor progression during follow-up [19]. Primary treatment failure was defined as failing to achieve complete treatment response according to mRECIST after up to two TACE or RFA. Local tumor progression (LTP) described by the appearance of tumor foci at the edge of the ablation zone, after at least one contrast-enhanced follow-up study has documented adequate ablation and an absence of viable tissue in the target tumor by using imaging criteria. This term applies regardless of when tumor foci were discovered either early or late in the course of imaging follow-up [36].

#### Survival

Secondary endpoints were to compare: *(i)* overall survival defined as time to last follow-up evaluation or death (patients with liver transplantation were censored at the date of transplantation) measured from the date of treatment; *(ii)* Progression-Free survival defines as the time interval between initial treatment and radiological progression (local or intra-hepatic distant recurrence).

#### Complications

Post-treatment morbi-mortality was collected and perioperative mortality was defined as death within 30 days of treatment. Morbidity was stratified as recommended by the Society of Interventional Radiology [37].

### Statistical analysis

To control selection bias and provide a more accurate matching on prognosis factor than using only propensity score we used one-to-one coarsened exact matching (CEM). Briefly, The idea of CEM is coarsen each variable into substantively meaningful groups on then to perform exact match on these coarsened data [38]. CEM was performed using three variables: Tumor size (categorized as < 20mm; 20-30mm and > 30mm); Serum AFP (categorized as < 10ng/ml; 10-100ng/ml and > 100ng/ml); and a propensity score variable including: age, sex, BMI, tumor number, platelet count and cirrhosis etiology. The propensity score variable was categorized with a caliper of 0.2. Tumor size and serum AFP were included separately in CEM to achieve exact matching on them because they are the main prognostic factors of survival and recurrence [24, 27, 30].

Data are expressed as mean (±standard deviation) or median (1^st^-3^rd^ quartiles) and compared using either the two-sample *t*-test or the Mann-Whitney test, according to data distribution. Percentages were compared using the Chi-2 test or Fisher's exact test. Survival without treatment failure and overall survival were computed by the Kaplan-Meier method and compared by the log-rank test. To identify factors associated with treatment failure; we first performed univariate analysis using univariate Cox regression. Variables with *p* < 0.1 were then introduced in a multivariate Cox Model and hazard ratios (HR) and corresponding 95% confidence intervals (CI) reported. Patients with primary treatment failure were censored at the date of the second treatment session. Two-sided statistical tests were used for all analyses. A *p*-value < 0.05 was considered as significant. Statistical analyses were performed with Stata 13.

## Abbreviations and acronyms

HCC: Hepatocellular carcinoma
SR: surgery resection
RFA: radiofrequency ablation
TACE: transarterial chemoembolization
CR: complete response
LTP: local tumor progression
CEM: Coarsened exact matching
